# Peroxiredoxin 4 Ameliorates T-2 Toxin-Induced Growth Retardation in GH3 Cells by Inhibiting Oxidative Stress and Apoptosis

**DOI:** 10.3390/molecules29235491

**Published:** 2024-11-21

**Authors:** Qirong Lu, Yi Zhu, Luyao Wang, Meng Mei, Yinsheng Qiu, Yu Liu, Shulin Fu, Jianglin Xiong, Pu Guo, Zhongyuan Wu, Xu Wang

**Affiliations:** 1 Hubei Key Laboratory of Animal Nutrition and Feed Science, School of Animal Science and Nutritional Engineering, Wuhan Polytechnic University, Wuhan 430023, China; qirongluvet@whpu.edu.cn (Q.L.); 18272347532@163.com (Y.Z.);; 2 State Key Laboratory of Biocatalysis and Enzyme Engineering, School of Life Sciences, Hubei University, Wuhan 430062, China; 3 National Reference Laboratory of Veterinary Drug Residues (HZAU) and MAO Key Laboratory for Detection of Veterinary Drug Residues, Huazhong Agricultural University, Wuhan 430070, China

**Keywords:** apoptosis, growth retardation, oxidative stress, peroxiredoxin 4, T-2 toxin

## Abstract

T-2 toxin, a highly toxic type A trichothecene, is a secondary fungal metabolite produced by various Fusarium species. The consumption of food and feed contaminated with T-2 toxin is a major factor contributing to growth retardation, posing significant risks to both human and animal health. However, the specific targets and mechanisms that mitigate T-2 toxin-induced growth retardation remain unclear. In this study, transcriptomic analysis was employed to identify key differentially expressed genes associated with the alleviation of T-2 toxin-induced growth retardation. Peroxiredoxin 4 (PRDX4), a gene linked to oxidative stress and apoptosis, was found to be one of the most downregulated in T-2 toxin-treated GH3 cells, an in vitro model of growth retardation. The experiments demonstrated that T-2 toxin significantly increased reactive oxygen species’ production, apoptosis, and cell cycle arrest while reducing the activity of antioxidant enzymes (superoxide dismutase and glutathione peroxidase) and PRDX4 expression in GH3 cells. Furthermore, PRDX4 silencing exacerbated T-2 toxin-induced oxidative stress and apoptosis, whereas PRDX4 overexpression effectively mitigated these effects. These findings highlight the protective role of PRDX4 in counteracting T-2 toxin-induced oxidative stress and apoptosis, suggesting that PRDX4 can serve as a therapeutic target for the treatment of T-2 toxin-induced growth retardation.

## 1. Introduction

T-2 toxin, primarily produced by various species of Fusarium, is a highly toxic type A trichothecene mycotoxin [1]. T-2 toxin contributes to the contamination of foods and feeds, causing mold and posing a major threat to both animal husbandry and human health [2]. It is commonly found in several cereal crops, including maize, soybeans, wheat, barley, oats, rye, and rice [3,4]. T-2 toxin has been detected in 92.6% of maize samples from Sichuan Province, China [5]; in up to 79.5% of feed samples from Shandong Province, China [6]; in 30.3% and 48.2% of feed samples from northern and eastern Europe, respectively [3]; in 65.0% of maize samples from New Zealand [7]; and in 55.91% of imported brands of 45 feline food and 48 canine food samples from the Chinese market produced in 2021–2022 [8]. It has also been detected in drinking water and traditional Chinese herbal medicines [9]. Numerous studies have shown that the consumption of food contaminated with T-2 toxin leads to a range of toxicological effects in both humans and animals, including damage to the cardiovascular system [10,11], immune system [12], digestive system [13], liver [14], skin [15], glomeruli [16] and reproductive system [17,18], as well as causing neurotoxicity [19,20] and growth retardation [21]. T-2 toxin is considered an emerging environmental threat because of its impact on growth retardation in animals, including Yangzhou goslings [22], juvenile Chinese mitten crabs [23], juvenile goats [24], broilers [25], and pigs [26]. Moreover, evidence from the literature suggests that T-2 toxin -induced growth retardation is associated with the increased production of reactive oxygen species (ROS), cell cycle arrest, apoptosis, and growth hormone deficiency [21,27,28,29]. However, the precise mechanisms by which T-2 toxin induces growth retardation remain unclear, necessitating further investigation into the molecular targets and signaling pathways involved.

Peroxiredoxin 4 (PRDX4), a member of the peroxiredoxin family, plays a critical role in regulating oxidative stress and apoptosis. The knockdown of PRDX4 exacerbated palmitic-acid-induced oxidative stress and cardiomyocyte apoptosis, while the overexpression of PRDX4 in H9C2 cells significantly alleviated these effects [30]. Similarly, the overexpression of PRDX4 reduced the oxidative stress and apoptosis induced by amyloid-beta oligomers (AβOs), whereas PRDX4 knockdown exacerbated these effects in HT-22 cells [31]. Furthermore, reducing PRDX4 expression significantly impaired the growth of glioblastoma multiforme cells and increased oxidative stress and apoptosis [32]. In PRDX4-knockout mice (PRDX4^−/−^), the increased expressions of oxidative-damage-related factors and elevated levels of apoptosis were observed in the ovaries compared with those of wild-type mice [33]. These studies suggest that PRDX4 exerts a protective effect against the oxidative stress and apoptosis caused by exogenous substances. 

Despite the established role of PRDX4 in modulating oxidative stress and apoptosis, its specific mechanisms in T-2 toxin-induced growth retardation have not been fully elucidated. The pituitary gland is an important endocrine organ in the body that can secrete growth hormone; the abnormal secretion of this hormone leads to growth retardation [34]. Hence, we used the rat pituitary tumor cell line GH3, widely used as a model for studying growth retardation [21,27,35], to investigate the mechanism of growth retardation caused by T-2 toxin. Therefore, this study was performed to evaluate the potential protective effect of PRDX4 in alleviating T-2 toxin-induced growth retardation, focusing on its role in mitigating oxidative stress and apoptosis. Our findings provide valuable insights into the molecular mechanisms underlying T-2 toxin-induced growth retardation and highlight PRDX4 as a potential therapeutic target for future research.

## 2. Results

### 2.1. Toxic Effect of T-2 Toxin on GH3 Cells

**Figure 1:**
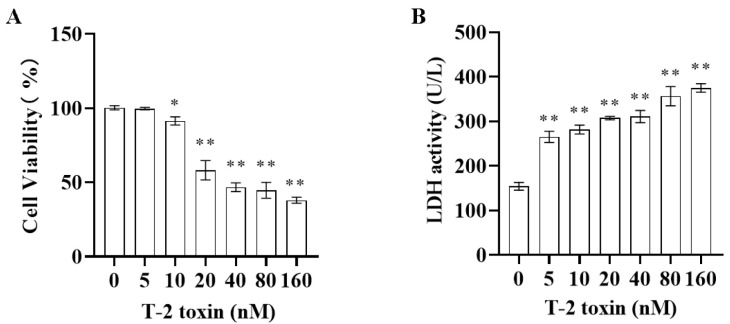
Cytotoxicity of T-2 toxin toward GH3 cells as measured using CCK-8 and LDH assays. GH3 cells were treated with 0–160 nM T-2 toxin for 24 h. (**A**) Cell viability was measured using the CCK-8 assay. (**B**) LDH activity in the cell supernatant was measured using the LDH assay. All data are presented as the average of three replicates. *p *< 0.05 (*) was defined as significant difference, *p *< 0.01 (**) was defined as extremely significant difference.

Both CCK-8 and LDH assays were employed to assess the cytotoxicity of T-2 toxin toward GH3 cells. The results indicated that after 24 h of incubation with 0–160 nM T-2 toxin, cell viability was significantly reduced (Figure 1A). Additionally, T-2 toxin at concentrations of 5–160 nM markedly increased the LDH activity in the cell supernatants (Figure 1B), demonstrating that T-2 toxin is significantly cytotoxic to GH3 cells. Consequently, for the subsequent experiments, GH3 cells were treated with 10 or 40 nM T-2 toxin to establish an in vitro growth retardation model.

### 2.2. Transcriptomic Analysis of Differentially Expressed Genes in T-2 Toxin-Treated GH3 Cells 

**Figure 2:**
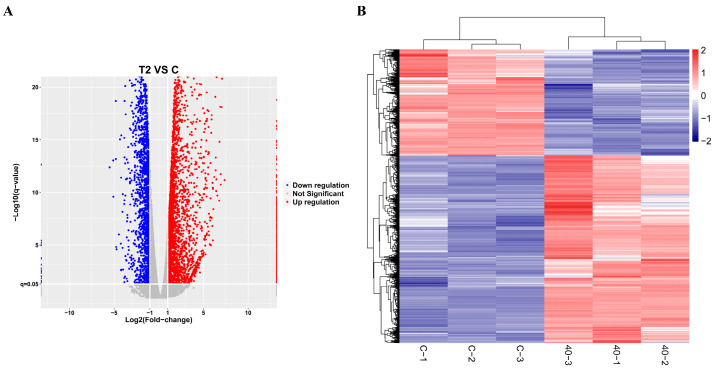
Transcriptomic analysis of differentially expressed genes in T-2 toxin-treated GH3 cells. (**A**) Volcano plot of differentially expressed genes. (**B**) Cluster analysis of differentially expressed genes.

**Figure 3:**
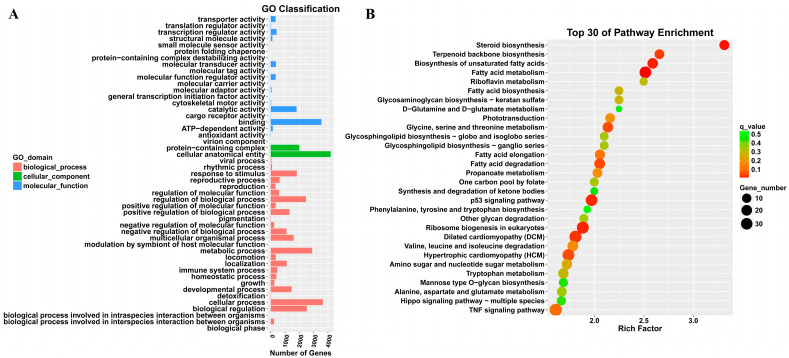
GO annotation and KEGG analysis of differentially expressed genes in T-2 toxin-treated GH3 cells. (**A**) GO annotation of differentially expressed genes. (**B**) KEGG analysis of differentially expressed genes.

Transcriptomics analysis was performed to explore the key differentially expressed genes involved in alleviating the growth retardation caused by T-2 toxin. In total, 5466 differentially expressed genes were identified, with 3486 significantly upregulated and 1980 significantly downregulated, including PRDX4 (Figure 2). Gene Ontology (GO) annotation and Kyoto Encyclopedia of Genes and Genomes (KEGG) analyses were used to examine the functions and signaling pathways of these genes. The GO classifications indicated molecular functions such as binding, catalytic activity, transcription regulation, and antioxidant activity, while the cellular components included protein complexes and virion components. The biological processes encompassed cellular processes, metabolic regulation, and growth and development (Figure 3A). The top 30 enriched pathways, including steroid biosynthesis, fatty acid metabolism, and the p53 signaling pathway, were also identified (Figure 3B). The enrichment of these pathways suggests that regulating them may help to prevent T-2 toxin-induced growth retardation, particularly by mitigating oxidative stress and apoptosis.

### 2.3. T-2 Toxin Reduced the Expression of PRDX4 in GH3 Cells and Induced Oxidative Stress and Apoptosis

**Figure 4:**
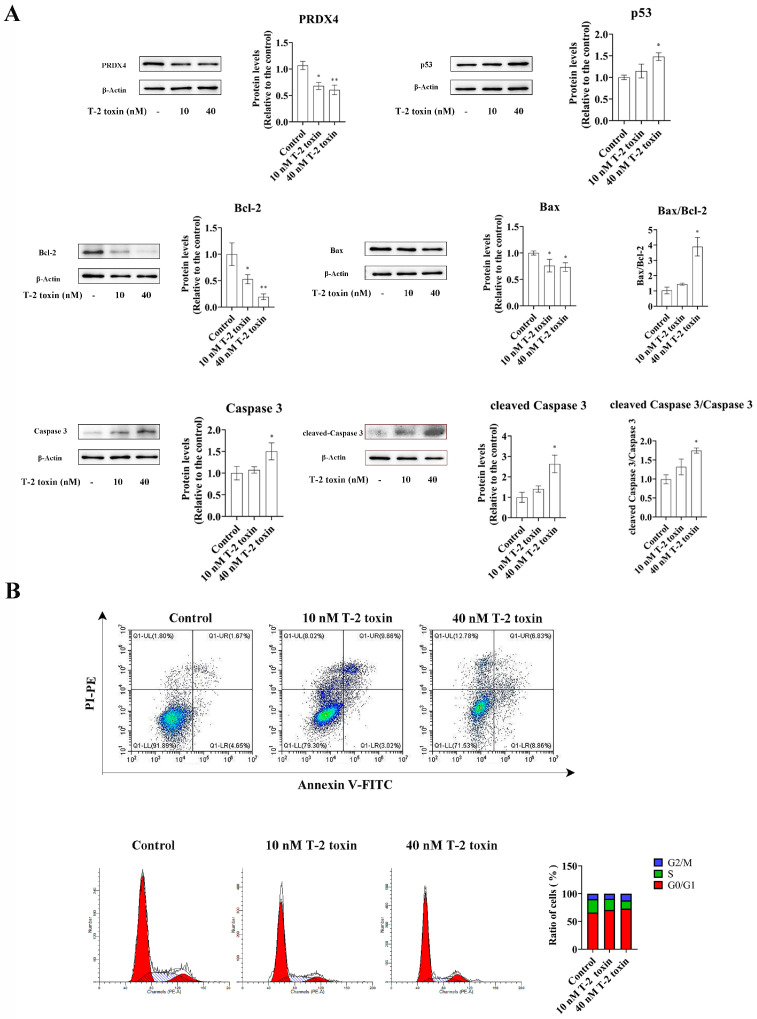
T-2 toxin induced growth retardation in GH3 cells by promoting apoptosis. (**A**) Immunoblotting of apoptosis-related proteins in T-2 toxin-treated GH3 cells. Protein density was quantified using ImageJ software (version 1.8.0). (**B**) Flow cytometry analysis of cell cycle and apoptosis in GH3 cells treated with T-2 toxin. All data are presented as the average of three replicates. *p *< 0.05 (*) was defined as significant difference, *p *< 0.01 (**) was defined as extremely significant difference.

**Figure 5:**
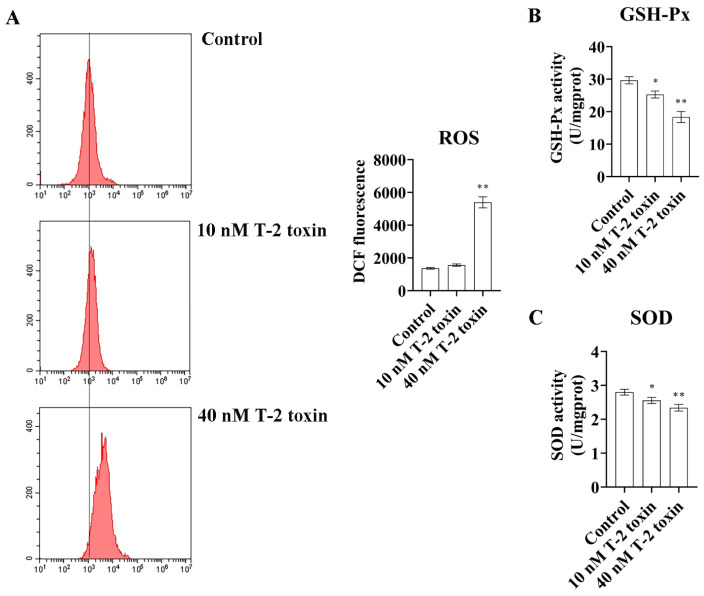
T-2 toxin induced growth retardation in GH3 cells by promoting oxidative stress. (**A**) Flow cytometry analysis of ROS content in GH3 cells treated with T-2 toxin. (**B**) The GSH-Px activities in GH3 cells treated with T-2 toxin was measured using GSH-Px assay kit. (**C**) The total SOD activities in GH3 cells treated with T-2 toxin was measured using the total SOD assay kit. All data are presented as the average of three replicates. *p *< 0.05 (*) was defined as significant difference, *p *< 0.01 (**) was defined as extremely significant difference.

Western blotting and flow cytometry analyses were conducted to investigate the impact of T-2 toxin on oxidative stress and apoptosis. After treatment with 10 and 40 nM T-2 toxin for 24 h, the expressions of apoptosis-related proteins (Bax/Bcl-2, p53, and cleaved caspase 3/caspase 3) were significantly increased compared with those of the control (0 nM T-2 toxin), while the PRDX4 protein expression decreased (Figure 4A). Flow cytometry further revealed that T-2 toxin significantly increased the apoptotic rate and caused cell cycle arrest in the G0/G1 and G2/M phases (Figure 4B). Additionally, T-2 toxin significantly elevated the ROS levels and reduced the total SOD and GSH-Px activities in GH3 cells (Figure 5). These results suggest that T-2 toxin induces apoptosis and oxidative stress, possibly due to PRDX4 downregulation in GH3 cells.

### 2.4. Overexpression of PRDX4 Alleviated T-2 Toxin-Induced Oxidative Stress and Apoptosis in GH3 Cells

**Figure 6:**
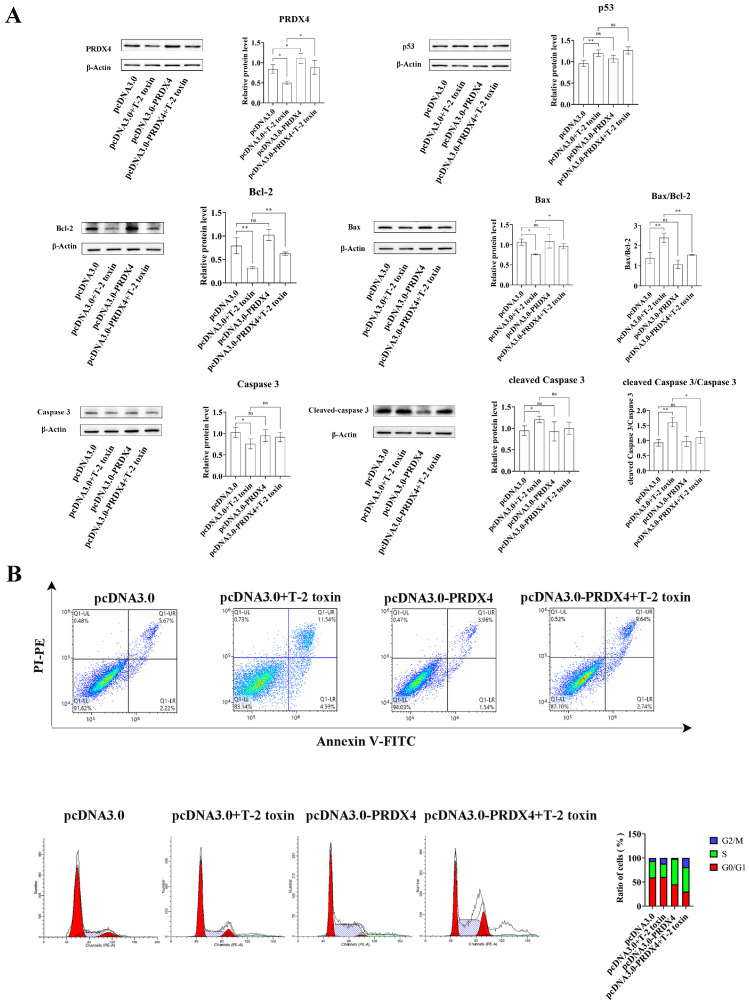
PRDX4 overexpression alleviates T-2 toxin-induced apoptosis. (**A**) Immunoblotting of apoptosis-related proteins in PRDX4-overexpressing and T-2 toxin-treated GH3 cells. Protein density was quantified using ImageJ software (version 1.8.0). (**B**) Flow cytometry analysis of cell cycle and apoptosis in GH3 cells treated with T-2 toxin and PRDX4 overexpression. All data are presented as the average from three replicates. *p *< 0.05 (*) was defined as significant difference, *p *< 0.01 (**) was defined as extremely significant difference.

**Figure 7:**
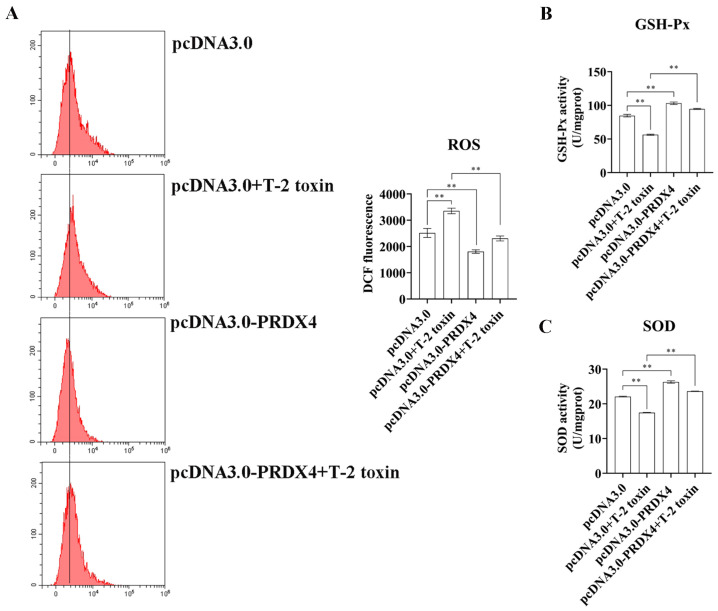
PRDX4 overexpression alleviates T-2 toxin-induced oxidative stress. (**A**) Flow cytometry analysis of ROS content in GH3 cells treated with T-2 toxin and PRDX4 overexpression. (**B**) The GSH-Px activities in GH3 cells treated with T-2 toxin and PRDX4 overexpression were measured using a GSH-Px assay kit. (**C**) The total SOD activities in GH3 cells treated with T-2 toxin and PRDX4 overexpression were measured using a total SOD assay kit. All data are presented as the average of three replicates. *p *< 0.01 (**) was defined as extremely significant difference.

To explore the protective role of PRDX4 against T-2 toxin-induced oxidative stress and apoptosis, PRDX4 was overexpressed in GH3 cells treated with T-2 toxin. Compared with cells treated with T-2 toxin and a control plasmid, PRDX4 overexpression significantly increased PRDX4 protein levels. The overexpression of PRDX4 also reduced the expression of apoptosis-related proteins such as Bax/Bcl-2 and cleaved caspase 3/caspase 3, although there was no significant difference in the p53 protein expression between the groups (Figure 6A). The flow cytometry results demonstrated that PRDX4 overexpression significantly decreased the accumulation of cells in the G0/G1 phase and reduced the levels of ROS compared with those for cells treated with T-2 toxin and the control plasmid (Figure 6B and Figure 7A). Additionally, the activities of GSH-Px and total SOD were measured to evaluate the antioxidant response. PRDX4 overexpression combined with T-2 toxin treatment significantly increased the GSH-Px and total SOD activities compared with those in cells treated with T-2 toxin and the control plasmid (Figure 7B,C). These results collectively indicate that PRDX4 plays a vital role in reducing the oxidative stress and apoptosis induced by T-2 toxin in GH3 cells. The overexpression of PRDX4 significantly alleviates the cytotoxic effects of T-2 toxin by enhancing antioxidant enzyme activity and inhibiting apoptosis, suggesting that PRDX4 could be a therapeutic target for mitigating the toxic effects of T-2 toxin.

### 2.5. Silencing of PRDX4 Exacerbated T-2 Toxin-Induced Oxidative Stress and Apoptosis in GH3 Cells

**Figure 8:**
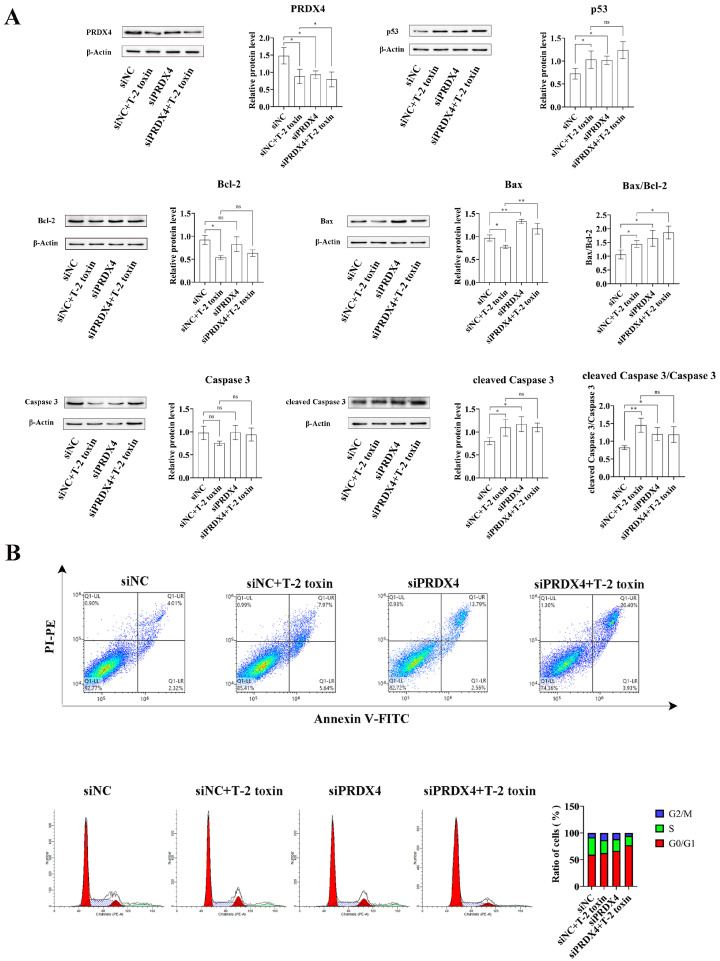
PRDX4 siRNA accelerates T-2 toxin-induced apoptosis. (**A**) Immunoblotting of apoptosis-related proteins in PRDX4 siRNA-transfected and T-2 toxin-treated GH3 cells. Protein density was quantified using ImageJ software (version 1.8.0). (**B**) Flow cytometry analysis of cell cycle and apoptosis in GH3 cells treated with T-2 toxin and PRDX4 siRNA. All data are presented as the average of three replicates. *p *< 0.05 (*) was defined as significant difference, *p *< 0.01 (**) was defined as extremely significant difference.

**Figure 9:**
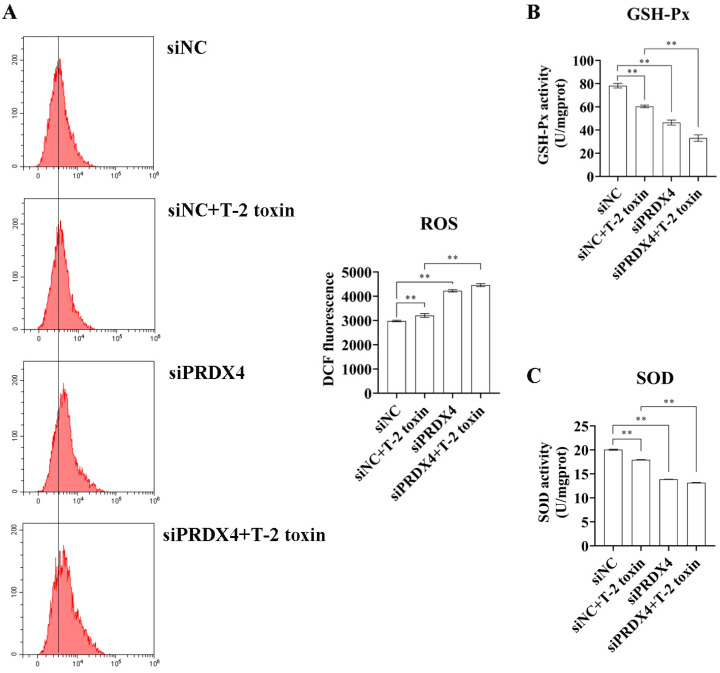
PRDX4 siRNA accelerates T-2 toxin-induced oxidative stress. (**A**) Flow cytometry analysis of ROS content in GH3 cells treated with T-2 toxin and PRDX4 siRNA. (**B**) The GSH-Px activities in GH3 cells treated with T-2 toxin and PRDX4 siRNA were measured using a GSH-Px assay kit. (**C**) The total SOD activities in GH3 cells treated with T-2 toxin and PRDX4 siRNA were measured using a total SOD assay kit. All data are presented as the average of three replicates. *p *< 0.01 (**) was defined as extremely significant difference.

To further investigate the relationship between PRDX4 and T-2 toxin-induced oxidative stress and apoptosis, PRDX4 siRNA was applied to GH3 cells treated with T-2 toxin. Compared with cells treated with T-2 toxin and control siRNA, cells treated with PRDX4 siRNA combined with T-2 toxin showed significantly decreased PRDX4 protein expression and increased expressions of apoptosis-related proteins, including p53, Bax/Bcl-2, and cleaved caspase 3/caspase 3 (Figure 8A). Additionally, PRDX4 siRNA combined with T-2 toxin significantly increased the apoptotic rate and led to a marked accumulation of cells in the G0/G1 and G2/M phases (Figure 8B). Furthermore, PRDX4 siRNA combined with T-2 toxin significantly elevated the ROS levels and reduced the activity of total SOD and GSH-Px in GH3 cells (Figure 9). These findings confirm that silencing PRDX4 exacerbates T-2 toxin-induced oxidative stress and apoptosis in GH3 cells.

## 3. Discussion

**Figure 10:**
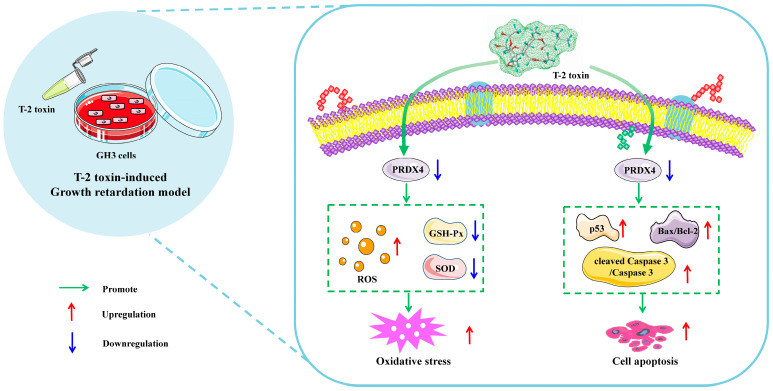
Schematic representation of the proposed mechanism by which T-2 toxin induces growth retardation in GH3 cells. T-2 toxin significantly reduces PRDX4 expression, induces oxidative stress by increasing the ROS content and decreasing the total SOD and GSH-Px activities, and induces apoptosis by increasing p53, cleaved caspase 3/caspase 3, and the Bax/Bcl-2 ratio. These findings highlight PRDX4’s protective role in mitigating the oxidative stress and apoptosis caused by T-2 toxin in GH3 cells.

The global contamination of food and feed by T-2 toxin poses a serious threat to both human and animal health [2]. The consumption of T-2 toxin-contaminated feed is a major factor contributing to growth retardation in animals. Numerous studies have shown that oxidative stress and apoptosis play critical roles in T-2 toxin-induced growth retardation, although the precise mechanisms remain unclear. By referencing previous studies [21], we selected GH3 cells to establish an in vitro model of T-2-toxin-induced growth retardation. PRDX4, a key regulator of oxidative stress and apoptosis [36], was identified as a gene of interest, making it clinically significant to investigate its role in T-2 toxin-treated GH3 cells. Our transcriptomic analysis revealed that PRDX4, a gene associated with oxidative stress, was one of the most downregulated genes in T-2 toxin-treated GH3 cells. The subsequent validation experiments demonstrated that PRDX4 effectively alleviated oxidative stress and apoptosis in this growth retardation model (Figure 10), suggesting that PRDX4 could be a therapeutic target for addressing T-2 toxin-induced growth retardation.

Transcriptomics is a powerful tool for investigating the health effects of exogenous substances and understanding disease mechanisms [37]. In this study, we identified 5466 differentially expressed genes in T-2 toxin-treated GH3 cells, including PRDX4. Given that growth retardation is closely linked to energy metabolism and oxidative stress [38], the functional classification of these genes revealed their involvement in ATP-dependent activity, antioxidant activity, and metabolic processes. Previous research has shown that T-2 toxin can increase mitochondrial ROS generation and ATP levels, leading to apoptosis in GH3 cells [28]. The KEGG enrichment analysis in this study revealed pathways including steroid biosynthesis, fatty acid metabolism, the p53 signaling pathway, and ribosome biogenesis. Ribosomes are a key target of T-2 toxin, as the toxin binds to them and inhibits protein synthesis, leading to growth retardation [2]. Additionally, the p53 pathway plays a crucial role in T-2 toxin-induced mitochondrial dysfunction and apoptosis in chondrocytes [39]. These results highlight that both oxidative stress and apoptosis are key contributors to T-2 toxin-induced growth retardation.

Oxidative stress and apoptosis are critical in T-2 toxin-induced growth retardation. Previous research has shown that T-2 toxin significantly induces cell cycle arrest and increases apoptosis in a growth retardation model [21]. In Chinese mitten crabs (Eriocheir sinensis), T-2 toxin exposure increased the expression of the apoptotic enzyme caspase while reducing the expressions of the antioxidant enzyme catalase (CAT) and the apoptosis inhibitor XIAP in the hepatopancreas [40]. Similarly, in porcine kidney cells (PK-15), T-2 toxin reduced the activity of antioxidant enzymes such as SOD, GSH-Px, and CAT while promoting ROS production and increasing the percentage of apoptotic cells [41]. In Sertoli cells (TM4), T-2 toxin induced oxidative stress by increasing the ROS content, inhibiting CAT and SOD activities, and promoting apoptosis [42]. Additionally, T-2 toxin causes G1-phase cell cycle arrest, intracellular-ROS-mediated oxidative stress, and apoptosis in GH3 cells [29]. These studies collectively demonstrate that T-2 toxin induces oxidative stress and apoptosis in growth retardation models. Similar results were observed in the current study. Compared with the control group, T-2 toxin treatment significantly increased ROS production, apoptosis rates, and G0/G1-phase cell cycle arrest while decreasing the activities of the antioxidant enzymes SOD and GSH-Px. However, the key targets and mechanisms in T-2 toxin-induced oxidative stress and apoptosis with regard to growth retardation still require further investigation.

PRDX4 has been identified as a key target for regulating oxidative stress and apoptosis in many studies [43]. In PRDX4-knockout mice, marked body weight loss and oxidative damage were observed in a dextran sulphate sodium-induced colitis model [44]. In a PRDX4^−/−^ mouse model, the deficiency in PRDX4 led to accelerated ovarian aging by increasing the apoptosis of granulosa cells [45], and PRDX4 knockout also increased oxidative damage and apoptosis in the ovaries [33]. In contrast, transgenic mice that expressed human PRDX4 showed reduced oxidative stress and inflammation in old age [46]. Moreover, silencing of PRDX4 increased hydrogen peroxide production and inflammation in renal cells [47]. In addition, the overexpression of PRDX4 reduced AβO-induced oxidative stress and apoptosis, while PRDX4 knockdown exacerbated these effects in HT-22 cells [31]. Similarly, PRDX4 knockdown significantly increased the rate of apoptosis and ROS generation in primary ectopic endometrial stromal cells [48]. Although the role of PRDX4 in T-2 toxin-induced growth retardation was not previously reported, our transcriptomics results and the known regulatory role of PRDX4 in oxidative stress and apoptosis prompted further investigation. In this study, we found that silencing PRDX4, in combination with T-2 toxin, significantly induced oxidative stress and apoptosis, while the overexpression of PRDX4 alleviated these effects. These findings suggest that PRDX4 could serve as a therapeutic target for the prevention and treatment of oxidative stress and apoptosis in T-2 toxin-induced growth retardation.

There are some limitations to the current study. Although PRDX4’s role was validated in vitro, other important proteins and pathways involved in growth retardation were not investigated in both in vitro and in vivo models. Moreover, the protective roles of PRDX4 have not yet been confirmed in animal models of T-2 toxin exposure. Research on the relationship between T-2 toxin, oxidative stress, and apoptosis in growth retardation is still in its early stages. Future studies focusing on antioxidants and antiapoptotic agents targeting PRDX4 are warranted.

## 4. Materials and Methods

### 4.1. Reagents and Chemicals

T-2 toxin (CAS No. 21259-20-1, HY-N6792) and dimethyl sulfoxide (CAS No. 67-68-5, DMSO, D8371) were purchased from MedChemExpress (Monmouth Junction, NJ, USA) and Solarbio (Beijing, China), respectively.

### 4.2. Cell Culture and Treatment

GH3 cells were acquired from the National Infrastructure of Cell Line Resource, Beijing, China. The cells were cultured in DMEM medium (Cytiva, Marlborough, MA, USA, SH30022.01) supplemented with 1% penicillin–streptomycin solution (Gibco, Thermo Fisher Scientific, Waltham, MA, USA, 15070063) and 10% fetal bovine serum (Vazyme Biotech, Nanjing, China, F101). The cells were incubated in a humidified incubator at 37 °C with 5% carbon dioxide. After incubation in six-well plates for 24 h, the GH3 cells were treated with T-2 toxin (10 or 40 nM) for 24 h [1].

### 4.3. Determination of Cell Viability

GH3 cells were seeded in 96-well plates. Following T-2 toxin treatment, the cell viability was analyzed using a Cell Counting Kit-8 (CCK-8) assay (Vazyme Biotech, Nanjing, China, A311), according to the manufacturer’s instructions and the literature [10]. In addition, cell supernatants were collected after T-2 toxin treatment to measure the lactate dehydrogenase (LDH) activity using an LDH assay kit (Nanjing Jiancheng Bioengineering Institute, Nanjing, China, A020-2), following the manufacturer’s instructions and the literature [11]. The absorbance values at 450 nm were measured using a microplate reader (SpectraMax i3x; Molecular Devices, Shanghai, China). 

### 4.4. Transcriptomic Analysis of T-2 Toxin-Treated GH3 Cells

The GH3 cell samples were divided into two groups: a control group (GH3 cells not treated with 40 nM T-2 toxin, n = 3) and a treated group (GH3 cells treated with 40 nM T-2 toxin, n = 3). After 24 h of incubation, the GH3 cells were harvested and sent to Shanghai Biotechnology Corporation, China, for transcriptome sequencing. The RNA-sequencing library was prepared according to Illumina’s standard instructions (VAHTS Universal V6 RNA-seq Library Prep Kit for Illumina^®^). An Agilent 4200 bioanalyzer was used to evaluate the concentration and size distribution of the cDNA library before sequencing on an Illumina NovaSeq 6000 (Illumina, San Diego, CA, USA). High-throughput sequencing was conducted following the manufacturer’s protocol (Illumina). Significantly differentially expressed genes were identified based on the false discovery rate value threshold (Q-value ≤ 0.05) and |log2FC| ≥ 1 using edgeR software 4.0.

### 4.5. Silencing and Overexpression of PRDX4 in GH3 Cells

Negative-control siRNA and PRDX4 siRNA were purchased from GenePharma Biotechnology (GenePharma, Shanghai, China, 104991). Full-length coding sequences of rat PRDX4 were cloned into a pcDNA3.0 vector. The siRNA and plasmids were transfected into GH3 cells using Lipo8000™ transfection reagent (Beyotime, Shanghai, China, C0533), following the manufacturer’s instructions and the literature [21].

### 4.6. Flow Cytometric Analysis of Cell Cycle and Apoptosis

GH3 cells were treated with T-2 toxin and/or transfected with the PRDX4 plasmid, or they were transfected with PRDX4 siRNA followed by incubation with T-2 toxin. After treatment, for the cell cycle assay, the GH3 cells were processed according to the manufacturer’s instructions and the literature [21] using a commercial cell cycle detection kit (KeyGEN BioTECH, Nanjing, China, KGA512) and analyzed using flow cytometry (Becton, Dickinson and Company, Franklin Lakes, NJ, USA). For the apoptosis assay, the GH3 cells were treated using an Annexin V-FITC/PI Apoptosis Detection Kit (Vazyme Biotech, Nanjing, China, A211), in accordance with the manufacturer’s instructions and the literature [21], and analyzed using flow cytometry (Beckman Coulter, Brea, CA, USA).

### 4.7. Measurement of ROS, Superoxide Dismutase (SOD), and Glutathione Peroxidase (GSH-Px)

GH3 cells were treated with T-2 toxin and/or transfected with the PRDX4 plasmid and then incubated with T-2 toxin; alternatively, they were transfected with PRDX4 siRNA and then incubated with T-2 toxin. To measure the intracellular ROS levels, the GH3 cells were processed using a commercial ROS assay kit (Beyotime, Shanghai, China, S0033S), in accordance with the manufacturer’s instructions and the literature [1], and analyzed using flow cytometry (Beckman Coulter, Brea, CA, USA).

For the measurement of SOD and GSH-Px levels, GH3 cells were treated with total SOD and GSH-Px assay kits (Nanjing Jiancheng Bioengineering Institute, Nanjing, China, A001-3 and A005-1), respectively, in accordance with the manufacturer’s instructions and the literature [28], and analyzed using a microplate reader (USCN Life Science, Wuhan, China).

### 4.8. Western Blotting Analysis

After the treatment of GH3 cells, Western blotting was performed according to the literature [28]. Briefly, the cells were lysed using RIPA buffer (Beyotime, Shanghai, China, P0013B) for 30 min on ice, followed by sonication and centrifugation at 12,000 rpm for 10 min at 4 °C. The supernatant was collected, and protein quantification was performed. A total of 20 μg of protein was separated through electrophoresis on 10% sodium dodecyl sulphate–polyacrylamide gel electrophoresis gels (Sangon Biotech, Shanghai, China, C671102) and transferred to polyvinylidene fluoride membranes (Cytiva, Marlborough, MA, USA, 10600023). The membranes were blocked with 5% skimmed milk and incubated with the primary antibody overnight at 4 °C. After thorough washing with TBST, the membranes were incubated with a horseradish-peroxidase-labeled secondary antibody (ABclonal, Wuhan, China, AS014). After further washing with TBST, exposure was performed using enhanced chemiluminescence reagents (ABclonal, Wuhan, China, RM00021) with a FluorChem E system (ProteinSimple, San Jose, CA, USA). Densitometry analysis was carried out using ImageJ software (National Institutes of Health, Bethesda, MD, USA), with β-actin (Proteintech, Wuhan, China, 81115-1-RR) used as a loading control for normalization.

### 4.9. Statistical Analysis

All the data are presented as the means ± standard deviations. Statistical analysis was conducted using one-way analysis of variance followed by Duncan’s post hoc analysis with SPSS version 18.0 (SPSS Inc., Chicago, IL, USA). A *p*-value of <0.05 was considered statistically significant.

## 5. Conclusions

Through transcriptomics and cell validation experiments, we confirmed the protective role of PRDX4 in counteracting oxidative stress and apoptosis in a growth retardation model, providing a potential target for treating T-2 toxin-induced growth retardation and providing a scientific basis for the development of inhibitors based on targets for the clinical treatment and prevention of T-2 toxin-induced growth retardation.

## Data Availability

The data presented in this study are available in the article.

## Abbreviations

AβO: amyloid-beta oligomers; Bax, Bcl2-associated X protein; CAT, catalase; CCK-8, Cell Counting Kit-8; DMSO, dimethyl sulfoxide; GO, Gene Ontology; GSH-Px, glutathione peroxidase; KEGG, Kyoto Encyclopedia of Genes and Genomes; LDH, lactate dehydrogenase; PRDX4, peroxiredoxin 4; ROS, reactive oxygen species; SOD, superoxide dismutase.
